# Palpable signs of skull fractures on physical examination and depressed skull fractures or traumatic brain injuries on CT in children

**DOI:** 10.1007/s00431-024-05807-w

**Published:** 2024-10-10

**Authors:** Silvia Bressan, Daniel Tancredi, Charles T. Casper, Liviana Da Dalt, Nathan Kuppermann

**Affiliations:** 1 Department of Women’s and Children’s Health, University of Padova, Via Giustiniani 3, 35128 Padova, Italy; 2 Departments of Emergency Medicine and Pediatrics, School of Medicine, University of California, Davis, Sacramento, CA USA; 3 Department of Pediatrics, University of Utah School of Medicine, Salt Lake City, UT USA

**Keywords:** Children, Head trauma, Skull fracture, Emergency medicine

## Abstract

To assess the actual presence of underlying depressed skull fractures and traumatic brain injuries (TBI) on computed tomography (CT) in children with and without palpable skull fractures on physical examination following minor head trauma. This was a secondary analysis of a prospective, observational multicenter study enrolling 42,412 children < 18 years old with Glasgow Coma Scale scores ≥ 14 following blunt head trauma. A palpable skull fracture was defined per the treating clinician documentation on the case report form. Skull fractures and TBIs were determined on CT scan by site radiologists. Palpable skull fractures were reported in 368/10,698 (3.4%) children < 2 years old, and in 676/31,613 (2.1%) of older children. Depressed skull fractures on CT were observed in 56/273 (20.5%) of younger children with palpable skull fractures and in 34/3047 (1.1%) of those without (rate difference 19.4%; 95%CI 14.6–24.2%), and in 30/486 (6.2%) vs 63/11,130 (0.6%) of older children (rate difference 5.6%; 95%CI 3.5–7.8%). TBIs on CT were found in 73/273 (26.7%) and 189/3047 (6.2%) of younger children with and without palpable skull fractures (rate difference 20.5%; 95%CI 15.2–25.9), and in 61/486 (12.6%) vs 424/11,130 (3.8%) of older children (rate difference 8.7%; 95%CI 6.1–12.0).

*Conclusions*: Although depressed skull fractures and TBIs on CT are more common in children with palpable fractures than those without, most of these children do not have underlying depressed fractures. The discriminatory ability of the scalp examination could be enhanced by direct bedside visualization of the skull, such as through ultrasound.

**Table:** 

**What is Known:**
• “*Palpable skull fractures” are a high-risk predictor in the PECARN traumatic brain injury (TBI) rule for children younger than 2 years.*
• *The reliability of physical exam findings suggestive of underlying skull fractures is unclear.*
**What is New:**
• *In children younger than 2 years of age, approximately one in five of those with “palpable skull fractures” will actually have a depressed skull fracture on CT, while 60% will have any skull fracture.*
• *The discriminatory ability of the scalp examination could be enhanced by direct bedside visualization of the skull, such as through ultrasound.*

## Introduction

Young children often present to the emergency department (ED) for signs of trauma to the head following minor blunt trauma. While signs of scalp trauma are most commonly minor and not concerning, in some instances, they can be signs of underlying skull fractures. On occasion, a depressed skull fracture can be palpated on physical examination of the scalp. Suspicion for depressed skull fractures is enhanced by a depression or step-off felt when the scalp is palpated. Depressed skull fractures are the result of a more intense direct impact to the head during trauma, thus increasing the risk of intracranial injuries. In addition, elevation of the depressed segment may be pursued depending on the level of depression below the inner table of adjacent bone. [1]

Suspicion of depressed skull fractures on physical examination of the scalp has indeed shown to be associated with higher risks of traumatic brain injuries (TBI) in children [2–5] and a CT scan is recommended in children younger than 2 years of age with “palpable skull fractures,” as per the Pediatric Emergency Care Applied Research Network (PECARN) rule-based algorithm [2]. However, a suspected skull fracture palpated on physical examination of the scalp often does not correspond to a depressed skull fracture on imaging. In addition, the interobserver agreement for signs of “palpable skull fractures,” although adequate, is suboptimal (kappa index of 0.67, with a lower 95% confidence interval limit of 0.41). [6]

Therefore, it is important to understand to what extent physical examination of the scalp is reliable in detecting actual depressed skull fractures. This information will help define the potential for point of care ultrasound (POCUS) in refining the accuracy of physical examination, by scanning the scalp to identify underlying fractures and define fractures characteristics. [7–9]

To our knowledge no studies have accurately described the frequency of depressed skull fractures and TBIs on computed tomography (CT) in children with “palpable skull fractures” on physical examination.

We aimed to determine the frequency of depressed skull fractures and TBIs on CT in children with and without physical findings suspicious for skull fractures on palpation of the scalp. As a secondary objective, we aimed to describe the frequency of any underlying skull fracture, and the type of TBIs associated with palpable signs of skull fractures on physical examination.

## Methods

### Study design and setting

This was a secondary analysis of the public use dataset from the PECARN prospective observational head trauma cohort study conducted in 25 EDs in the USA [2]. Data were collected between June 2004 and September 2006. The parent study was approved by the Human Subjects Research Committee at each site. Full details of the parent study methods have been described previously [1]. This secondary analysis qualified as non-human subject research because the data used were from a de-identified dataset that can be accessed and downloaded at https://pecarn.org/datasets/. As such, it was deemed exempt by the University of California Davis institutional review board. We followed the Strengthening the Reporting of Observational Studies in Epidemiology (STROBE) guidelines [10].

### Selection of participants

Participants enrolled in the main cohort study were younger than 18 years presenting to the ED within 24 h of head trauma with Glasgow Coma Scale (GCS) scores of 14 to 15. Children with trivial head trauma (defined as ground level falls or running into stationary objects and no evidence of TBI other than scalp abrasions or lacerations) and patients with bleeding disorders or ventricular shunts were excluded. Patients with penetrating head trauma, pre-existing neurologic disease impairing clinical assessment, or syncope or seizures preceding the head trauma and patients transferred to the ED with neuroimaging already obtained were also excluded from the parent study.

### Definitions

Suspicion of a palpable skull fracture was defined based on the case report form of the PECARN parent study [2]. In the PECARN TBI clinical prediction rule, palpable skull fractures marked as “yes” or “unclear examination” (due to scalp swelling) were combined for the predictor “palpable skull fracture.” We followed this definition for the current analysis. In addition, in the presence of a palpable skull fracture, the case report form asked whether the fracture felt depressed, with response options being no, yes, or unclear exam.

### Outcomes

The outcomes for this study were defined as follows:

1. Depressed skull fractures were defined as any depressed or displaced skull fractures as reported in the CT report by a site or study radiologist.
2. Any skull fracture on CT, including linear non-complicated skull fractures, either associated with TBIs or in isolation.
3. TBIs on CT, for the purpose of the present study, were defined as any acute traumatic intracranial findings (excluding isolated skull fractures).
4. Clinically important TBIs (ciTBI) were defined, as per the parent study, as death from the TBI, TBIs requiring neurosurgical procedures (i.e., intracranial pressure monitoring, elevation of depressed skull fracture, ventriculostomy, hematoma evacuation, lobectomy, tissue debridement, dura repair, other), intubation for at least 24 h for the TBI, or hospitalization for 2 or more nights because of ongoing signs or symptoms in association with TBI on CT. The criterion of at least 2 nights of hospitalization was determined by consensus of investigators and defined to exclude brief intubation for imaging or overnight admission for minor or questionable CT findings. In the parent study, research coordinators or site investigators reviewed the medical records of hospitalized patients to determine the presence of ciTBI. For patients discharged from the ED, standardized surveys were delivered via telephone between 7 and 90 days after the ED visit. In addition, medical records were reviewed to identify any children with missed TBIs. For patients unreachable by telephone or mail, research staff reviewed the patient’s medical records, ED process improvement records, trauma registries, and county morgue records to help ensure that no patients with ciTBIs were missed [2].

CT scans were obtained at the treating clinicians’ discretion and were interpreted by site faculty radiologists unaware of the clinical findings documented on the case report forms. For inconclusive scans, a central study pediatric radiologist made definitive interpretations without knowledge of clinical data [2]. Patients could meet the definition for more than one outcome.

### Statistical analysis

We described categorical variables using counts and proportions. We reported continuous variables as medians and interquartile ranges (IQR). We calculated the frequencies and proportions of outcomes and exact binomial confidence intervals (CIs) for the proportions in children with and without palpable skull fractures. We compared the prevalence of outcomes between groups using rate differences with 95%CI. We used the Agresti-Caffo confidence intervals that correct for the poor coverage of Wald confidence intervals for risk differences, by adding one success and one failure to each group before determining the Wald confidence interval endpoints [11]. We also described and compared the prevalence of outcomes between children with palpable skull fractures marked as “yes” or “unclear exam (e.g., scalp swelling impedes exam)” on the case report forms. We conducted separate analyses for children younger than 2 years, as in the PECARN TBI rule a “palpable skull fracture” was a high-risk predictor in this age group only.

All analyses were performed using SAS University Edition (SAS Institute, Cary, NC).

## Results

### Characteristics of study participants

**Fig. 1:**
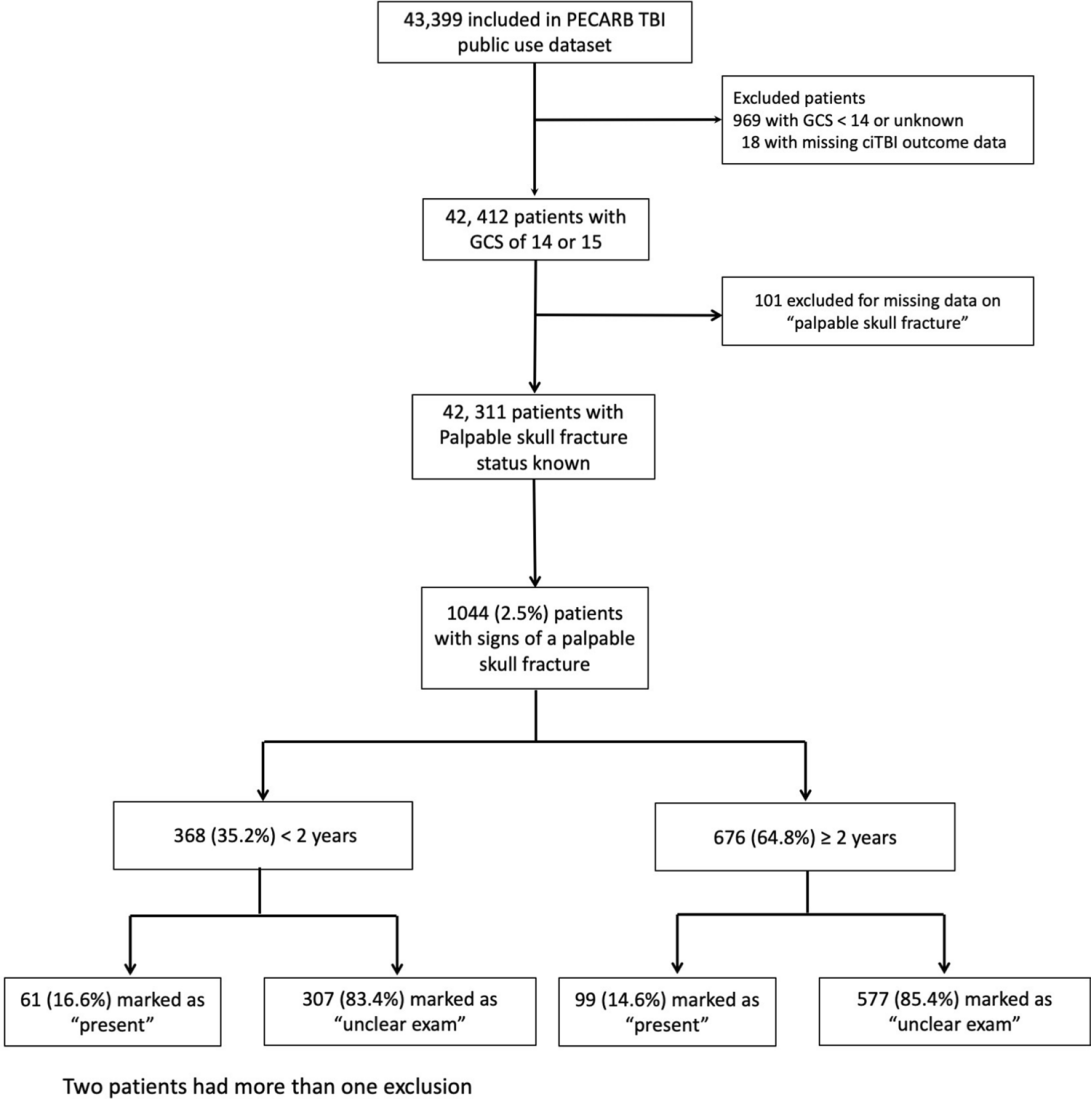
Study profile. GCS, Glasgow Coma Scale; PECARN, Pediatric Emergency Applied Research Network; TBI, traumatic brain injury

Of the 42,311 eligible children, 1044 (2.5%) were reported to have palpable signs of skull fractures on physical examination, of which 160 (15.3%) were marked as “yes” and 884 (84.7%) as “unclear exam.” Palpable signs of skull fractures were reported in 368 of 10,698 (3.4%) children younger than 2 years and in 676 of 31,613 (2.1%) of older children (Fig. 1).

Within the category of palpable signs of skull fractures marked as “yes,” clinicians reported that the fractures clearly felt depressed in 39 of the 160 (24.4%) cases (28 of 61 in children younger than 2 years and 11 of 99 in older children). The clinical characteristics of children with and without palpable signs of skull fractures are reported in Tables 1 and 2 for children younger than 2 years and those aged 2 years and older, respectively. Younger children with palpable signs of skull fractures more often had altered mental status, non-frontal scalp hematomas, and abnormal behavior per guardian’s report. These children also more often sustained severe mechanisms of injury, compared with children without palpable skull fractures (Table 1). Children aged 2–18 years with palpable signs of skull fractures more often had altered mental status, concurrent signs of basilar skull fractures, severe headaches, and more often sustained severe mechanisms of injury, compared with children without palpable signs of skull fractures (Table 2).

**Table 1:** Characteristics of children with and without “palpable skull fractures”* on physical examination for children < 2 years

	Children with palpable skull fractures (*n* = 368)	Children without palpable skull fractures (*n* = 10,330)
Age, median (IQR), years	0.0 (0.0–1.0)	1.0 (0.0–1.0)
Male	206/368 (56.0%)	5670/10,330 (54.9%)
Mechanism of injury		
Fall from elevation	197/352 (56.0%)	5597/10,239 (54.7%)
Fall down stairs	49/352 (13.9%)	1458/10,239 (14.2%)
Fall to ground from standing/walking/running	22/352 (9.4%)	935/10,239 (9.1%)
Walked or run into stationary object	18/352 (5.1%)	578/10,239 (5.6%)
Object struck head accidental	14/352 (4.0%)	503/9502 (4.9%)
Occupant in motor vehicle collision	7/352 (2.0%)	247/10,239 (2.4%)
Assault	4/352 (1.1%)	66/10,239 (0.6%)
Other	30/352 (8.5%)	855/10,239 (8.3%)
Concomitant significant injury to other part of the body	10/367 (2.7%)	262/10,303 (2.5%)
Other PECARN ciTBI predictors		
Altered mental status	86/365 (23.6%)	1120/10,267 (10.9%)
Loss of consciousness > 5 s	7/368 (1.9%)	453/10,330 (4.4%)
Non-frontal scalp hematoma	214/364 (58.8%)	1473/10,232 (14.4%)
Acting abnormally per guardian	77/342 (22.5%)	1359/9935 (13.7%)
Severe mechanism of injury	117/352 (33.2%)	2219/10238 (21.7%)

* ciTBI*, clinically important Traumatic Brain Injuries; *IQR*, interquartile range; *PECARN*, Pediatric Emergency Care Applied Research Network

^*^ “Palpable skull fractures” includes children for whom the case report forms were marked as either “yes” or “unclear exam” for signs of skull fracture based on palpation of the scalp

**Table 2:** Characteristics of children with and without “palpable skull fractures”* on physical examination for children 2–18 years

	Children with palpable skull fractures (*n* = 676)	Children without palpable skull fractures (*n* = 30,934)
Age, median (IQR), years	8.0 (4.0–13.0)	8.0 (4.0–13.0)
Male	474/676 (70.1%)	19,994/30,934 (64.6%)
Mechanism of injury		
Fall from elevation	108/672 (16.1%)	5745/30,765 (18.7%)
Fall down stairs	33/672 (4.9%)	1311/30,765 (4.3%)
Fall to ground from standing/walking/running	68/672 (10.1%)	3616/30,765 (11.8%)
Walked or run into stationary object	38/672 (6.7%)	1797/30,765 (5.8%)
Object struck head accidental	56/672 (8.3%)	2541/30,765 (8.3%)
Occupant in motor vehicle collision	58/672 (8.6%)	3401/30,765 (11.1%)
Assault	86/672 (12.8%)	2817/30,765 (9.2%)
Other	225/672 (33.5%)	9537/30,765 (31.0%)
Concomitant significant injury to other part of the body	101/674 (15.0%)	3895/30830 (12.6%)
PECARN ciTBI predictors		
Altered mental status	149/668 (22.3%)	4122/30708 (13.4%)
Signs of basilar skull fracture	22/671 (3.3%)	208/30,689 (0.7%)
Any loss of consciousness	143/676 (21.2%)	5591/30,937 (18.1%)
Severe headache	37/610 (6.1%)	766/29643 (2.6%)
History of vomiting	80/670 (11.9%)	3904/30,733 (12.7%)
Severe mechanism of injury	138/672 (20.5%)	3770/30736 (12.3%)

*ciTBI*, clinically important Traumatic Brain Injuries; *IQR*, interquartile range; *PECARN*, Pediatric Emergency Care Applied Research Network

^*^ “Palpable skull fractures” includes children for whom the case report forms were marked as either “yes” or “unclear exam” for signs of skull fracture based on palpation of the scalp

### Study outcomes

CT scans were performed on 759 (72.7%) of 1044 children with palpable signs of skull fractures compared with 14,177 (34.4%) of 41,267 patients without these physical signs. CT scan rates were similar between children younger than 2 years and older children with palpable signs of skull fractures (273/368 (74.2%), and 486/676 (71.9%), respectively).

The study outcomes are detailed in Table 3 for children with and without palpable signs of skull fractures, by age group. All study outcomes were more frequent in children with palpable signs of skull fractures compared to those without, with greater rate differences in the younger age group.

**Table 3:** Outcomes in children with and without “palpable skull fractures”* on physical examination

Children < 2 years			
	No./No (%) (95%CI)		
Outcome	Children with palpable skull fractures (*n*=368)	Children without palpable skull fractures (*n*=10,330)	Rate difference (95% CI)
*Depressed skull fracture on CT* (90 of 3320 CT scans)	56/273 (20.5%) (15.7–25.3)	34/3047 (1.1%) (0.7–1.5)	19.4% (14.8, 24.4)
*Any skull fracture on CT* (449 of 3320 CT scans)	161/273 (59.0%) (52.9–64.9)	288/3047 (9.5%) (8.4–10.6)	49.5% (43.5, 55.3)
*TBI on CT (excluding isolated skull fractures)* (282 of 3320 CT scans)	73/273 (26.7%) (21.6–32.4)	189/3047 (6.2%) (5.4–7.1)	20.5% (15.4, 26.0)
*Clinically important TBI* (98 of 10,698)	34/368 (9.2%) (6.5–12.7)	64/10,330 (0.6%) (0.5–0.8)	8.6% (5.8, 11.8)
Death	0 / 368 (0.0%) (0.0–1.0)	0 / 10328 (0.0%) (0.0–0.04)	0.0% (–0.3, 0.8)
Neurosurgery	12/368 (3.3%) (1.7–5.6)	7/10330 (0.07%) (0.0–0.1)	3.2% (1.6, 5.3)
Intubation > 24 hours for TBI	2/368 (0.5%) (0.1–2.0)	1/10330(0.01%) (0.0–0.1)	0.5% (–0.1, 1.7)
Hospitalization > 2 nights for TBI	36/368 (9.8%) (7.0–13.3)	78/10330 (0.8%) (0.6–0.9)	9.0% (6.2, 12.3)
Children 2–18 years			
	No./No (%) (95%CI)		
Outcome	Children with palpable skull fractures (*n*=676)	Children without palpable skull fractures (*n*=30,937)	Rate difference (95%CI)
*Depressed skull fracture on CT* (93 of 11,616 CT scans)	30/486 (6.2%) (4.0–8.3)	63/11,130 (0.6%) (0.4–0.7)	5.6% (3.6, 7.9)
*Any skull fracture on CT* (477 of 11,616 CT scans)	75/486 (15.4%) (12.3–19.0)	402/11,130 (3.6%) (3.3–4.0)	11.8% (8.7, 15.2)
*TBI on CT (excluding isolated skull fractures)* (485 of 11,616 CT scans)	61/486 (12.6%) (9.7–15.8)	424/11,130 (3.8%) (3.5–4.2)	8.7% (5.9, 11.9)
*Clinically important TBI* (278 of 31,610)	39/676 (5.8%) (4.1–7.8))	239/30,937 (0.8%) (0.7–0.9)	5.0% (3.3, 6.9)
Death	0/676 (0.0%) (0.0–0.5)	0/30935 (0.06–0.1%) (0.0–1.2)	0.0% (–0.1, 0.4)
Neurosurgery	12/676 (1.8%) (0.9–3.1)	29/30,937 (0.1%) (0.1–0.1)	1.7% (0.8, 2.9)
Intubation > 24 hours for TBI	0/676 (0.0%) (0.0–0.5)	5/30,935 (0.02%) (0.01–0.04)	0.0% (–0.2, 0.4)
Hospitalization > 2 nights for TBI	42/676 (6.2%) (4.5–8.3)	313/30,936 (1.0%) (0.9–1.1)	5.2% (3.5, 7.2)

*”Palpable skull fractures” includes children for whom the case report forms were marked as either “yes” or “unclear exam” for signs of skull fracture based on palpation of the scalp

Rate differences are reported with 95% Agresti-Caffo Confidence Interval

Within the group of children with palpable signs of skull fractures marked as “yes,” CT scans were performed in 149/160 (93.1%) compared to 610/884 (69.0%) of those classified as having “unclear exams.” CT scan rates were similar between children younger than 2 years and older children with palpable signs of palpable skull fractures marked as “yes” (57/71 (93.4%), and 92/99 (92.9%), respectively).

Table 4 reports the outcome frequency of children with palpable signs of palpable fractures marked as “yes” and those marked as “unclear exam,” by age group. All study outcomes were more frequent in children with palpable signs of skull fractures marked as “yes” compared to those with “unclear exams,” with greater rate differences in the younger age group.

**Table 4:** Outcomes in children with palpable skull fractures on physical examination marked as “yes” versus “unclear exam”

Children < 2 years			
	No./No (%) (95%CI)		
Outcome	Children with palpable skull fractures “yes” (*n*=61)	Children with palpable skull fractures “unclear exam” (*n*=307)	Rate difference (95%CI)
*Depressed skull fracture on CT* (56 of 273 CT scans)	28/57 (49.1%) (35.6–62.7)	28/216 (13.0%) (8.8-18.2)	36.2% (22.3, 49.4)
*Any skull fracture on CT* (161 of 273 CT scans)	44/57 (77.2%) (64.2–87.3)	117/216 (54.2%) (47.3-60.9)	23.0% (9.4, 34.9)
*TBI on CT (excluding isolated skull fractures)* (73 of 273 CT scans)	18/57 (31.6%) (19.9–45.2)	55/216 (25.5%) (19.8-31.8)	6.1% (–6.7, 19.8)
*Clinically important TBI* (34 of 368)	14/61 (23.0%) (13.2–35.5)	20/307 (6.5%) (4.0–9.9)	16.4% (7.1, 28.7)
Death	0/61 (0.0%) (0.0–5.9)	0/307 (0.0%) (0.0–1.2)	0.0% (–1.9, 4.4)
Neurosurgery	7/61 (11.5%) (4.7–22.2)	5/307 (1.6%) (0.5–3.8)	9.8% (2.4, 19.1)
Intubation > 24 hours for TBI	2/61 (3.3%) (0.4–11.4)	0/307 (0%) (0.01.2)	3.3% (–0.9, 9.7)
Hospitalization > 2 nights for TBI	14/61 (23.0%) (13.2–35.5)	22/307 (7.2%) (4.6–10.7)	15.8% (5.4, 27.3)
Children 2–18 years			
	No./No (%) (95%CI)		
Outcome	Children with palpable skull fractures “yes” (*n*=99)	Children with palpable skull fractures “unclear exam” (*n*=577)	Rate difference (95%CI)
*Depressed skull fracture on CT* (30 of 486 CT scans)	14/92 (15.2%) (8.3–25.5)	16/394 (4.1%) (2.3–6.6)	11.2% (4.0, 19.3)
*Any skull fracture on CT* (75 of 486 CT scans)	20/92 (21.7%) (13.8–31.6)	55/394 (14.0%) (10.7–17.8)	7.8% (–0.9, 17.3)
*TBI on CT* (61 of 486 CT scans)	12/92 (13.0%) (6.9–26.1)	49/394 (12.4%) (9.3–16.1)	0.6% (–6.5, 8.9)
*Clinically important TBI* (39 of 676)	11/99 (11.1%) (5.7–19.0)	28/577 (4.9%) (3.3–6.9)	6.3% (0.3, 13.4)
Death	0/99 (0.0%) (0.0–3.7)	0/577 (0.0%) (0.0–0.6)	0.0% (–1.1, 2.8)
Neurosurgery	4/99 (4.0%) (1.1–10.0)	8/569 (1.4%) (0.6–2.7)	2.6% (–1.0, 7.7)
Intubation > 24 h for TBI	0/99 (0.0%) (0.0–3.7)	0/577 (0.0%) (0.0–0.6)	0.0% (–1.1, 2.8)
Hospitalization > 2 nights for TBI	12/99 (12.1%) (6.4–20.2)	30/577 (5.2%) (3.5–7.3)	6.9% (0.7, 14.3)

Rate differences are reported with 95% Agresti-Caffo Confidence Interval

The type of TBIs on CT of all children with palpable skull fractures on physical examination are reported in Table 5.

**Table 5:** TBI on CT of children with signs of palpable skull fractures

TBI in patients who underwent CT (*n* = 759)	Children < 2 y *n* = 273 No. (%)^a^	Children ≥ 2 y *n* = 486 No. (%)^a^
Subdural hematoma	80 (29.3%)	110 (22.6%)
Extra-axial hematoma^b^	62 (22.7%)	71 (14.6%)
Subarachnoid hemorrhage	61 (22.3%)	90 (18.5%)
Cerebral hemorrhage	33 (12.1%)	81 (16.7%)
Epidural hematoma	38 (13.9%)	60 (12.3%)
Cerebral contusion	29 (10.6%)	123 (25.3%)
Pneumocephalus	17 (6.2%)	130 (26.7%)
Cerebellar hemorrhage	2 (0.7%)	9 (18.5%)
Midline shift	18 (6.6%)	35 (7.2%)
Diastasis	18 (6.6%)	26 (5.3%)
Cerebral edema	3 (1.1%)	33 (6.8%)
Intraventricular hemorrhage	7 (2.6%)	10 (2.1%)
Shear injury/diffuse axonal injury	1 (0.4%)	12 (2.5%)
Traumatic infarction/sigmoid sinus thrombosis	0 (0.0%)	2 (0.4%)

^a^Patients could have more than 1 TBI on CT

^b^Intracranial hemorrhages that were not further categorized as subdural hematomas, epidural hematomas, or subarachnoid hemorrhage

## Discussion

In this secondary analysis of a large prospectively populated pediatric minor head trauma database, we found that children with palpable signs of skull fractures on examination of the scalp represented a minority of the population, but they underwent head CT scans in approximately 70% of cases. CT scan rates were more than 90% when excluding palpable signs of skull fractures marked as “unclear exam” on the case report forms. Furthermore, although children with palpable skull fractures were found to have higher frequencies of depressed skull fractures, any skull fracture on CT, TBI on CT, and ciTBI than those without palpable signs, most did not have these outcomes. This shows that clinicians overestimate the presence of depressed skull fractures when examining children with minor head trauma. In children younger than 2 years of age approximately only one in five of those with “palpable skull fractures” will actually have a depressed skull fracture on CT, while 60% will have any skull fracture. At all ages, however, higher percentages of all study outcomes were found in children with palpable signs of skull fractures marked as “yes,” compared to those defined as having “unclear exam.” It is intuitive that higher clinicians’ confidence of their clinical examination would be associated with greater diagnostic discriminatory ability, thus justifying higher CT rates in children defined as having clear signs of palpable skull fractures.

The frequencies of our study outcomes were higher for children < 2 years old with palpable signs of skull fractures compared to children 2–18 years old, both overall, and with respect to perceived reliability of scalp physical examination findings (i.e., findings marked as “yes” versus “unclear exam”). These results are consistent with the parent study and are related with the different anatomical characteristics of the skull and scalp in younger children. The thinner calvarium, more vascularized scalp and looser subcutaneous tissues facilitate deformation and fractures of the skull and the development of large scalp hematomas as a result of direct trauma in younger children [12]. In both age groups, patients with palpable signs of skull fractures more often presented with other associated PECARN TBI rule predictors compared to children without these signs. This may reflect the higher head impact sustained by children who have skull fractures, thus explaining a higher frequency of associated symptoms.

Differently from our work, a recent secondary analysis of a multicentre prospective study including 1,018 children with blunt head trauma (of whom 85 (8.3%) had any skull fracture on CT, and 18 (1.8%) had depressed or basilar skull fractures) showed that physical examination was highly specific, but poorly sensitive in identifying skull fractures [13]. However, this study only included children who underwent head CT; the analysis grouped together depressed and basilar skull fractures and did not analyze separately younger and older children. Participants included children with GCS scores of any severity, with 72% reported to have GCS scores of 15. These reasons likely explain the lower clinician sensitivity found in that study compared to ours.

Other large multicenter prospective studies found that suspected depressed skull fractures on scalp examination was a predictor of TBIs in children, thus recommending a head CT scan in children with these physical examination findings [4, 5, 14]. However, the above studies grouped the findings of suspected depressed fractures either with penetrating skull injuries or tense fontanelle [5], or with suspected open fractures [4], and overall included fewer than 200 patients with these findings in each study. In addition, these studies did not separately analyze those patients with unclear scalp findings due to the presence of overlying large and boggy scalp hematomas as we did in this study. Large and/or boggy scalp hematomas complicate the identification of a step-off of the skull on physical examination, with the diagnosis more challenging and less reliable.

Skull ultrasound has proven to be accurate in identifying skull fractures underlying scalp hematomas [8, 9, 12, 15, 16] and, most importantly, can define their characteristics (depressed or complex fractures) [7]. POCUS could thus be a valuable adjunct to refine clinical decision-making in children with palpable signs of skull fractures according to the PECARN TBI rule. In those children with unclear physical findings of skull fractures and no other age-based high-risk PECARN predictors, the absence of fractures detected on skull ultrasound may support clinicians’ decision to opt for close ED observation rather than for a head CT. In contrast, the detection of a depressed skull fracture on ultrasound would prompt the performance of a head CT scan.

Our data, however, do not provide assistance about the best clinical course when a linear uncomplicated skull fracture is detected on ultrasound in otherwise asymptomatic children. Older reports have demonstrated that skull fractures identified on plain skull radiographs are associated with significantly higher rates of TBIs both in children and adult head trauma patients, although up to 50% of TBIs can occur in the absence of skull fractures [17–20]. At the time of those studies, however, ultrasound was not routinely available or used as a bedside screening tool. Previous studies and guidelines thus recommended skull radiographs as a screening tool for TBIs in the assessment of young infants with large scalp hematomas as the sole manifestation of head trauma and recommended CT scans in infants with skull fractures identified on radiographs due to the higher risk of associated TBIs [21–24]. In the era of high-quality clinical prediction rules and patient-centered outcomes, better estimates of the risk of clinically important injuries are available for head-injured children. The most recent prospective multicenter prediction rule studies on pediatric head trauma, however, have not separately analyzed the association between linear uncomplicated skull fractures on CT scan and the presence of TBIs in otherwise asymptomatic children [2, 4, 5, 14]. On the other hand, a recent systematic review and meta-analysis has shown that children with isolated uncomplicated linear skull fractures on CT scan have essentially no risk of neurologic deterioration and can be safely discharged home if asymptomatic and in the absence of suspicion of non-accidental injury [25–27].

The results of our study must be interpreted in light of its limitations. First, other clinical findings on examination of the scalp, such as crepitus, and not only the palpation of a step-off of the skull, may have contributed to the classification of physical findings as palpable skull fractures by clinicians. Unfortunately, we could not explore this nuance in our dataset given how the data were collected. Second, not all children in the study received head CT scans. Our work, however, is based on a prospective observational study that was not meant to interfere with routine clinical decision-making, and mandating the performance of a CT scan in all participants with head trauma would have been unethical. Nonetheless, more than 70% of children classified as having palpable skull fractures on examination received a CT scan increasing the likelihood that our results accurately reflect those that would be obtained in the overall population of children with palpable signs of skull fractures in general practice. Third, although to our knowledge we used the largest available prospective pediatric head trauma database, our sample size of patients with scalp findings and skull fractures is still relatively small, as shown by the relatively wide 95% CI for some subgroup analyses. Fourth, nearly 20 years have elapsed since data collection and practice has changed over time. However, given the large rigorous prospectively collected database used for our secondary analysis, and the nature of the research question explored, this is highly unlikely to have influenced our findings. In addition, our study shows how publicly available datasets from high quality studies are useful to explore refinement in clinical management when new tools are introduced in clinical practice. Finally, our results do not apply to the definitions of suspected depressed skull fractures used in other clinical prediction rule studies and thus cannot be extended to clinical practice in settings using those rules [4, 5].

Despite these limitations, our findings have important implications for clinical practice. We demonstrated that children with palpable signs of skull fractures following minor head trauma have higher frequencies of depressed skull fractures and TBIs on CT than those without. However, the discriminatory ability of the scalp examination is suboptimal and could be enhanced by direct bedside visualization of fracture characteristics, such as through skull ultrasound.

## Data Availability

The de-identified dataset used for this paper can be accessed and downloaded at https://pecarn.org/datasets/.

## Abbreviations

CT: Computed tomography
ED: Emergency department
GCS: Glasgow Coma Scale
PECARN: Pediatric Emergency Applied Research Network
POCUS: Point of care ultrasound
TBI: Traumatic brain injury
